# Early rise in central venous pressure during a spontaneous breathing trial: A promising test to identify patients at high risk of weaning failure?

**DOI:** 10.1371/journal.pone.0225181

**Published:** 2019-12-05

**Authors:** Sebastián Dubo, Emilio Daniel Valenzuela, Andrés Aquevedo, Manuel Jibaja, Dolores Berrutti, Christian Labra, Rossana Lagos, María Fernanda García, Vanessa Ramírez, Milton Tobar, Fabricio Picoita, Cristian Peláez, David Carpio, Leyla Alegría, Carolina Hidalgo, Karen Godoy, Alejandro Bruhn, Glenn Hernández, Jan Bakker, Ricardo Castro

**Affiliations:** 1 Departamento de Kinesiología, Facultad de Medicina, Universidad de Concepción, Concepción, Chile; 2 Programa de Doctorado en Ciencias Médicas, Universidad de la Frontera, Temuco, Chile; 3 Departamento de Medicina Intensiva, Facultad de Medicina, Pontificia Universidad Católica de Chile, Santiago, Chile; 4 Unidad de Pacientes Críticos, Hospital Dr. Sótero del Río, Santiago, Chile; 5 Unidad de Cuidados Intensivos, Hospital Eugenio Espejo, Quito, Ecuador; 6 Escuela de Medicina, Universidad Internacional de Ecuador, Quito, Ecuador; 7 Centro de Terapia Intensiva, Hospital de Clínicas, Universidad de la Republica de Uruguay, Montevideo, Uruguay; 8 Programa de Doctorado en Ciencias Médicas, Pontificia Universidad Católica de Chile, Santiago, Chile; 9 Unidad de Cuidados Intensivos Cardioquirúrgicos, Hospital Guillermo Grant Benavente, Concepción, Chile; 10 Unidad de Cuidados Intensivos Neuroquirúrgicos, Hospital Guillermo Grant Benavente, Concepción, Chile; 11 Department of Pulmonary and Critical Care, Columbia University College of Physicians and Surgeons, New York, New York, United States of America; 12 Department of Intensive Care Adults, Erasmus MC University Medical Center, Rotterdam, Netherlands; 13 Department of Pulmonary and Critical Care, New York University Medical Center, New York, New York, United States of America; Scuola Superiore Sant’Anna, ITALY

## Abstract

**Background:**

The spontaneous breathing trial (SBT) assesses the risk of weaning failure by evaluating some physiological responses to the massive venous return increase imposed by discontinuing positive pressure ventilation. This trial can be very demanding for some critically ill patients, inducing excessive physical and cardiovascular stress, including muscle fatigue, heart ischemia and eventually cardiac dysfunction. Extubation failure with emergency reintubation is a serious adverse consequence of a failed weaning process. Some data suggest that as many as 50% of patients that fail weaning do so because of cardiac dysfunction. Unfortunately, monitoring cardiovascular function at the time of the SBT is complex. The aim of our study was to explore if central venous pressure (CVP) changes were related to weaning failure after starting an SBT. We hypothesized that an early rise on CVP could signal a cardiac failure when handling a massive increase on venous return following a discontinuation of positive pressure ventilation. This CVP rise could identify a subset of patients at high risk for extubation failure.

**Methods:**

Two-hundred and four mechanically ventilated patients in whom an SBT was decided were subjected to a monitoring protocol that included blinded assessment of CVP at baseline, and at 2 minutes after starting the trial (CVP-test). Weaning failure was defined as reintubation within 48-hours following extubation. Comparisons between two parametric or non-parametric variables were performed with student T test or Mann Whitney U test, respectively. A logistic multivariate regression was performed to determine the predictive value on extubation failure of usual clinical variables and CVP at 2-min after starting the SBT.

**Results:**

One-hundred and sixty-five patients were extubated after the SBT, 11 of whom were reintubated within 48h. Absolute CVP values at 2-minutes, and the change from baseline (dCVP) were significantly higher in patients with extubation failure as compared to those successfully weaned. dCVP was an early predictor for reintubation (OR: 1.70 [1.31,2.19], p<0.001).

**Conclusions:**

An early rise in CVP after starting an SBT was associated with an increased risk of extubation failure. This might represent a warning signal not captured by usual SBT monitoring and could have relevant clinical implications.

## Introduction

Mechanical ventilation (MV) is a life-saving intervention in intensive care units (ICUs). Its application, however, may be associated with serious complications including higher mortality and costs, often directly linked to its duration [1,2]. Consequently, earlier weaning from mechanical ventilation leads to substantial benefits from clinical and non-clinical perspectives [3]. Accordingly, timely weaning from MV represents a crucial process for every patient, since weaning failure is a determinant of the poor outcomes associated with longer duration of MV and longer ICU and hospital stay [4–6]. In the clinical routine, weaning is accomplished through a standardized process called the spontaneous breathing trial (SBT) which is a test of the real conditions of breathing without the ventilator, before performing the extubation.

The SBT assesses the risk of weaning failure by evaluating physiological responses to the workload imposed by discontinuing positive pressure ventilation during a period of 60 to 120 minutes [7–12]. However, the trial can be very demanding for some critically ill patients, inducing excessive physical and cardiovascular stress, and eventually cardiac dysfunction, arrhythmias, or ischemia [13–15]. In addition, a failed SBT may also cause muscle overload and fatigue. The latter is underscored by a recent study showing that a renewed one-hour of MV after a successful SBT reduced reintubation rate [16], supporting the idea that SBT itself imposes a non-negligible load and risks. Therefore, it appears as relevant to explore new potential variables, not captured by the usual SBT monitoring, that could identify patients at high risk of extubation failure before or during the first minutes of an SBT [6,9,17]. An early warning signal to stop the trial could not only avoid a prolonged and potentially harmful exercise, but also allow further evaluations and interventions in a timelier fashion.

Extubation failure with emergency reintubation is a serious adverse consequence of a failed weaning process. Reintubation may be required by 5 to 30% of critically ill who initially had a successful SBT and is eventually associated with higher mortality [18,19]. Moreover, some data suggest that as many as 50% of patients that fail weaning do so because of cardiac dysfunction [20,21]. Unfortunately, it is not easy to monitor cardiovascular function at the time of the SBT since invasive hemodynamic monitoring has usually been removed before the SBT, and echocardiography is not universally available. A recent study [21] found that a fluid unresponsive state before SBT was associated with higher probability of weaning-induced pulmonary edema. However, evaluation of fluid responsiveness in the pre-weaning context is not possible in every patient [22]. In addition, a parallel increase in pulmonary artery occlusion pressure and central venous pressure (CVP) has been observed in cases of weaning failure of cardiac origin [23,24].

In view of the above, we aimed at studying if central venous pressure (CVP) changes were related to weaning failure after starting an SBT. We hypothesized that an early rise on CVP could signal a cardiac failure when handling a massive increase on venous return following the discontinuation of positive pressure ventilation. This CVP rise could identify a subset of patients at high risk for extubation failure.

## Methods

### Study design

We performed a prospective multicenter observational study between July 2015 and September 2016 in four medical-surgical ICU in Chile and Ecuador. The study was approved by the Institutional Review Board of each center approved the study and waived informed consent due to the observational nature of the study (CEC-MED-UC 14–102, Pontificia Universidad Católica de Chile 4/7/2015; CEC Servicio de Salud Concepción, Chile 15-06-30, 7/15/2015; CEC Servicio de Salud Metropolitano Sur-Oriente, Santiago Chile 6/8/2015; Subdirección de Docencia e Investigación, Hospital Eugenio Espejo Quito, Ecuador, 5/1/2015). As the study was intended to rely on the usual clinical practice, the only specific study-related monitoring was a blinded standardized CVP assessment (see below). STROBE guidelines were used for reporting this study [25].

### Study population

This study was performed in critically ill patients on MV for ≥24h in whom the attending clinician had decided to initiate an SBT based on clinical criteria. Additional inclusion criteria were resolution or stabilization of the original disease, clinical stability on controlled MV (PaO_2_/FiO_2_ >150 with FiO_2_ ≤0.4, positive end-expiratory pressure (PEEP) ≤8 cmH_2_O and good cough reflex, and arterial pH ≥7.35) and a central venous catheter in place. Patients were excluded if age <18 years-old, had inadequate mentation or permanent neurological damage, non-controlled infection, hemodynamic instability and/or hypoperfusion, tracheostomy in situ, and do-not-resuscitate (DNR) status. Only the first SBT per patient was considered.

### Study protocol

Patients eligible for SBT were ventilated with 5 cmH_2_O of PEEP and pressure support (PS) before starting SBT to standardize measurements. Subsequently, the SBT was performed on a “T-piece” connected to an O_2_ source. The duration of SBT was 1 or 2 hours, as decided by attending physicians.

A successful SBT was determined by previously published criteria [12], whereas extubation was decided on an individual basis [4]. For the purposes of this study, weaning failure was defined as reintubation within 48-h following extubation [11]. No clinical follow-up was conducted after 48-h.

Besides usual clinical and laboratory variables, CVP was measured at baseline, at 2-minutes (CVP-test), and at the end of the SBT. CVP data collected for the study were blinded to the attending physicians. A unique researcher per center measured the CVP after previous training. Intra-observer variability was not assessed. CVP was assessed through a jugular or subclavian central venous catheter with the patient in the supine position at 45° [26]. Pressures were measured using a TruWave disposable pressure transducer (*Edwards Lifesciences*, *Irvine*, *CA*) that was zeroed at approximately 5 cm below the sternal angle, which roughly corresponds to the 4^th^ intercostal space in the mid-axillary line. The transducer and the position of the patient remained unchanged during the SBT. CVP measurement was performed at the base of the *c* wave at the end of expiration in patients on MV and during SBT, except in patients with evident expiratory efforts, where CVP was measured at the start of expiration.

### Statistical analysis

According with previous studies and our clinical experience, we estimated that 70–80% of patients would be extubated at the end of SBT, with a reintubation rate ranging from 5–30% [11,18]. Due to the observational nature and of this trial, our main consideration when estimating the sample size was to capture a relevant number of reintubation cases. Therefore, we aimed at including at least 180 patients for hypothesis testing.

Normality of the variables was tested by the Kolmogorov-Smirnov test. Parametric and non-parametric variables were expressed as mean ± standard deviation (SD) with median and interquartile range (IQR), respectively. Categorical variables were expressed as percentages. Comparisons between parametric and non-parametric variables were performed with t- test or Mann Whitney U-test, respectively. Categorical variables were compared using chi-square or Fisher’s exact test, as appropriate.

A logistic multivariate regression was performed to determine the predictive value of clinical variables at 2-min after starting the SBT, on extubation failure. Variables considered for the multivariate model were selected in a stepwise fashion. Variables that met *p* <0.2 on univariate analysis and relevant clinical variables were considered in the model. Odds ratio (OR) and 95% CI were calculated for age, APACHE II, MV days, ICU length of stay, central venous O_2_ saturation (S_cv_O_2_), heart rate, respiratory rate, and change in CVP (*d*CVP) at 2-minutes.

Statistical analyses were performed with SPSS, version 20 (IBM. Chicago Illinois, USA) and Stata 15 (*StataCorp*. College Station, TX). The level of significance was set at p-value <0.05 and 95% confidence intervals.

## Results

A total of 204 patients were recruited, but two patients were excluded due to incomplete data. After finishing the SBT, 165 patients were extubated, eleven of whom were reintubated within 48-h. The remaining 37 patients did not meet the clinical criteria to be extubated and were not included in this report. There were no differences at baseline in the demographic and clinical characteristics of patients that were successfully weaned versus those with extubation failure (Table 1).

**Table 1:** Demographic and clinical characteristics of the study patients.

	All	Weaning Success	Extubation failure
Patients, n	165	154	11
Age, (years)	53 ± 22	54 ± 21	47 ± 24
Male gender, (%)	111 (54)	86 (55)	9 (81)
Body Mass Index, (kg/m^2^)	27 ± 5	27 ± 6	25 ± 2
APACHE II	20 ± 8	20 ± 8	18 ± 10
SOFA on admission	9 ± 4	9 ± 4	8 ± 3
SOFA at the day of SBT	4 (2–6)	4 (2–6)	5 (2–6)
ICU length of stay (days)	6 (3–10)	6 (3–10)	6 (4–10)
MV-days before SBT	5 (3–9)	6 (3–9)	5 (4–10)
Admission diagnosis, n (%)			
Sepsis	92 (45)	67 (43)	3 (27)
Postsurgical	41 (20)	34 (22)	1 (9)
Trauma	17 (8)	14 (9)	1(9)
Neurosurgical	18 (9)	13 (8)	3 (27)
Other	35 (18)	28 (19)	3 (27)
Cardiac comorbidities; n (%)			
Heart Failure	29 (15)	26 (17)	3 (27)
Coronary Artery Disease	14 (9)	12 (18)	2 (18)

Continuous variables are expressed as mean ± SD or median (interquartile range), and categorical variables are expressed as number (percentage).

*APACHE* Acute Physiology and Chronic Health Evaluation, *SOFA* Sequential Organ Failure Assessment, *SBT* spontaneous breathing trial, *ICU* intensive care unit, *MV* mechanical ventilation. No statistically significant differences between groups.

**Fig 1:**
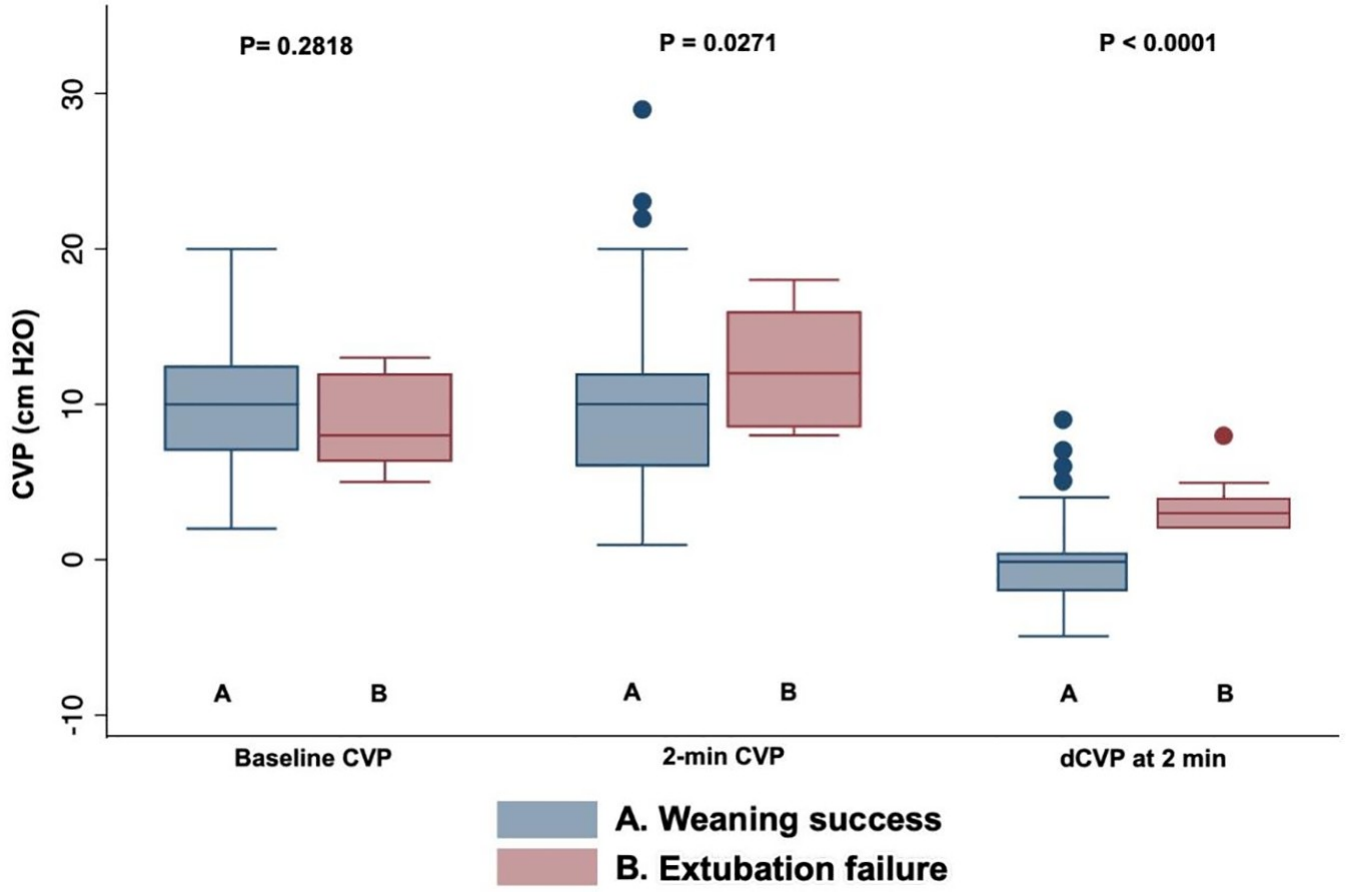
Box-plots comparing CVP values between patients with weaning success versus extubation failure at baseline and after 2-minutes of starting SBT (CVP-test).

Both, absolute CVP values at 2-minutes, and the change in CVP from baseline (*d*CVP) were significantly higher in patients with extubation failure as compared to those successfully weaned (Table 2 and Fig 1). Baseline and 2-minute values for heart rate, systolic blood pressure, respiratory rate, arterial and S_a_O_2_ are also shown in Table 2. At the end of the SBT, no variable–including CVP–showed a significant difference between groups (Table 3).

**Table 2:** Clinical variables at baseline and at 2 minutes of the spontaneous breathing trial.

	Baseline	2 min	*p* value^a^
Heart Rate (beats/min)			
Weaning Success	86 ± 18	87 ± 18	<0.001
Extubation failure	96 ± 18	94 ± 13	0.52
Systolic arterial blood pressure (mmHg)			
Weaning Success	132 (121–151)	137 (121–156)	0.001
Extubation failure	136 (130–146)	130 (123–159)	0.42
CVP (mmHg)			
Weaning Success	10 (7–13)	10 (6–12)	0.018
Extubation failure	8 (6–12)	12 (9–16)	0.001
dCVP (mmHg)			
Weaning Success		0 (-2-0.5)	<0.0001§
Extubation failure		3 (2–4)	
Respiratory Rate (breaths/min)			
Weaning Success	18 (15–22)	20 (17–24)	<0.001
Extubation failure	18 (13–25)	19 (17–24)	0.08
S_a_O_2_ (%)			
Weaning Success	96 (95–98)	97 (95–99)	0.33
Extubation failure	97 (95–98)	96 (93–100)	0.83

Continuous variables are expressed as mean ± SD or median (interquartile range)

*SBT* spontaneous breathing trial, *CVP* central venous pressure, *dCVP* change in CVP from baseline to 2-minutes after starting SBT, *S*_*a*_*O*_*2*_ arterial oxygen saturation.

^*a*^*p* values represent statistical comparisons between variables comparing 2 min to baseline by paired t-test or Wilcoxon rank-sum test as appropriate.

^§^ p-value compares dCVP at 2 minutes between groups

**Table 3:** Clinical variables at the end of the spontaneous breathing trial.

	Weaning Success	Extubation failure	*p* value ^a^
Heart Rate (beats/min)	88 ± 17	98 ± 16	0.079
Systolic arterial blood pressure (mmHg)	139 (124–158)	134 (124–134)	0.765
CVP (mmHg)	10 (6–12)	10 (5–12)	0.782
Respiratory Rate (breaths/min)	21 (18–24)	19 (17–25)	0.989
Arterial oxygen saturation, S_a_O_2_ (%)	96 (94–99)	98 (94–100)	0.459
Central venous oxygen saturation, S_cv_O_2_ (%)	75 (69–79)	74 (61–79)	0.468

Continuous variables are expressed as mean ± SD or median (interquartile range)

*SBT* spontaneous breathing trial, *CVP* central venous pressure

^a^*p* values represent statistical comparisons between variables comparing groups by unpaired t-test or U-Mann Whitney test as appropriate

In the multivariate model, the only early (2-minutes) predictor for reintubation was *d*CVP (OR: 1.70 [1.31,2.19], *p* < 0.001). The other covariates did not reach statistical significance: age (OR: 0.98 [0.95,1.02], *p* = 0.472), MV days (OR: 0.99 [0.91,1.09], *p* = 0.913), S_cv_O_2_ (OR: 0.99 [0.97,1.21], *p* = 0.163), heart rate (OR: 0.99 [0.98,1.01], *p* = 0.941), and respiratory rate (OR: 0.99 [0.91,1.09], *p* = 0.927).

## Discussion

Our main finding was that an early rise in CVP predicts extubation failure during an SBT. In fact, a rise in CVP two minutes after starting the trial increased the risk of reintubation by 70%. At that time-point, no other clinical variable behaved in a similar way of predicting extubation failure.

CVP is a complex parameter subject to many measurement errors and biases, and interpretation difficulties [27]. CVP is determined by the interaction between cardiac and venous return curves, both of which can be affected by discontinuation of positive pressure ventilation. In patients with normal cardiac function and without fluid overload or strong inspiratory efforts, the normal CVP response during an SBT is usually a decrease [28]. In consequence, an early increase on CVP may be considered as abnormal in the SBT context.

Strong respiratory efforts resulting in negative pleural pressure swings have an important effect on CVP values. These interferences could have been captured by an esophageal balloon [29,30], but as this was a pragmatic and observational study, we did not include this measurement in the protocol. However, the CVP-test was assessed only 2 minutes after discontinuing positive pressure ventilation and therefore it is unlikely that this factor could have influenced CVP values in a clinically relevant fashion. For the same observational nature of our study, we cannot speculate on why the CVP signal was not present at the end of the SBT period but just almost immediately after the start of SBT. In any case, it still reinforces the potential value of the CVP rise as an early, strong but transient warning signal that should be identified promptly after starting the SBT when present.

Cardiac dysfunction is a frequent cause of extubation failure [21,23]. Particularly, overt or even silent systolic or diastolic dysfunction as well as coronary heart disease, arrhythmias and other heart disorders decrease the likelihood of successful weaning [13,15,21,28]. The transition from positive to negative intrathoracic pressures may create unfavorable loading conditions for the right heart due to a massive increase on venous return and a simultaneous increase in left ventricular afterload, which coupled with an increased adrenergic tone during the SBT [21,28] may induce cardiac dysfunction. In an early study, Lemaire et al [14] observed that a rise in CVP of 12 mmHg was associated with the development of pulmonary edema soon after the beginning of spontaneous breathing. Besides, Dres et al [23] found that patients who failed a SBT due to cardiac dysfunction, had a mean increase in CVP of 5 mmHg during the SBT whereas patients without weaning induced cardiac dysfunction had an increase of only 1 mmHg. In this latter study, patients who developed cardiac dysfunction during the SBT were preload unresponsive just before the start of the SBT. This suggests that these patients were already at the limit of their Starling’s cardiac function, in the flat part of that curve [31]. These findings are in accordance with Weil et al that proposed to use the CVP as a measure of venous return intolerance in the context of fluid resuscitation [32].

This latter consideration is also relevant since almost three-quarters of the included patients had sepsis, trauma or other inflammatory conditions, thus being probably exposed to the risk of fluid overload. In patients with fluid overload venous return may increase after discontinuing positive pressure ventilation leading eventually to weaning-induced cardiac dysfunction or pulmonary edema [23,24]. Liu et al [21] showed that some fluid-unresponsive patients that had a previous episode of weaning failure, were successfully weaned after moving to a preload dependent state with the use of diuretics or vasodilators. Even though fluid responsiveness was not assessed in this study we could speculate that a CVP-test looks after the same phenomena from another perspective.

Assessment of cardiac dysfunction in an SBT setting is complicated by the fact that pulmonary artery catheters or other cardiac output monitoring devices are usually no longer in place. In addition, echocardiography is difficult to perform in a sitting, sometimes rapidly breathing patient, and the technique might not be easily available in resource-limited settings. In this context, the CVP-test appears to be a potential useful monitor that is currently not included in classic SBT-related assessments. Then, a rise in CVP should be considered only as a warning signal as it does not provide diagnostic clues without additional evaluations.

Although our study is only observational and hypothesis-generating, our results suggest a potential clinical relevance in performing a CVP-test. An abnormal early rise in CVP after starting an SBT should promote a prompt cardiovascular assessment in some patients, allowing the establishment of unloading interventions such as diuretics, vasodilators, inodilators, etc., in selected patients. This could halt and even revert the weaning failure track the patient is undergoing into, as suggested by Rousti et al [33] in the same context of weaning from MV. These considerations could imply, as well, more appropriate use for specialized cardiovascular assessments as echocardiography or other methods to patients with an abnormal CVP-test.

Our study has many limitations. The measurements of CVP could have been subject to intra-observer variability although we tried to minimize this risk by using a rigorous standardized procedure. Second, in spite of CVP measurement standardization, changes in intrathoracic pressure due to increased respiratory rate or strong respiratory swings could have influenced CVP measurements. Third, the lack of bedside assessment of cardiac function and performance limits the inference of causality in this study. Fourth, we did not register data on fluid balances. Fifth, we did not perform follow-up assessments so we cannot provide data on subsequent weaning attempts after the first extubation failure. Sixth, very few patients with chronic obstructive pulmonary disease were included in this cohort, and thus, our results might not be applicable for that specific subset of patients.

In the end, as central venous catheters are still in place in most ventilated patients when and SBT is started, CVP can be easily assessed at the bedside. If confirmed by further studies, a CVP-test might represent a new strategy to identify patients at high risk of failure only minutes after starting a SBT. In addition, this may allow clinicians to order additional evaluations or interventions to improve the probabilities of weaning success.

## Conclusions

An early rise in CVP after starting a spontaneous breathing trial was associated with an increased risk of extubation failure. This might represent a warning signal not captured by classic SBT monitoring and could have relevant clinical implications. Given the limitations of this current study, these findings need to be explored and confirmed by further studies.
